# More than Just Antioxidants: Redox-Active Components and Mechanisms Shaping Redox Signalling Network

**DOI:** 10.3390/antiox11122403

**Published:** 2022-12-04

**Authors:** Monika Kuczyńska, Patrycja Jakubek, Agnieszka Bartoszek

**Affiliations:** Faculty of Chemistry, Gdańsk University of Technology, 80-233 Gdańsk, Poland

**Keywords:** redox homeostasis, cellular signalling, redox network, reductive stress, antioxidants

## Abstract

The concept of oxidative stress as a condition underlying a multitude of human diseases has led to immense interest in the search for antioxidant-based remedies. The simple and intuitive story of “the bad” reactive oxygen species (ROS) and “the good” antioxidants quickly (and unsurprisingly) lead to the commercial success of products tagged “beneficial to health” based solely on the presence of antioxidants. The commercial success of antioxidants by far preceded the research aimed at understanding the exact redox-related mechanisms that are in control of shaping the states of health and disease. This review describes the redox network formed by the interplay of ROS with cellular molecules and the resulting regulation of processes at the genomic and proteomic levels. Key players of this network are presented, both involved in redox signalling and control of cellular metabolism linked to most, if not all, physiological processes. In particular, this review focuses on the concept of reductive stress, which still remains less well-established compared to oxidative stress.

## 1. Introduction

The health-promoting properties of bioactive substances present in plant-based foods have become the subject of an impressive amount of research in the field of food science. Antioxidant phytochemicals attracted the attention of scientists and the public already in the 1990s, when the role of reactive oxygen species (ROS) in the pathogenesis of non-infectious chronic diseases, popularly known as civilisation diseases, began to be understood. Research on the molecular basis of chronic diseases such as atherosclerosis, hypertension, diabetes, and cancer, has led to the conclusion that all these diseases have a common risk factor, namely the disruption of cellular redox homeostasis called *oxidative stress* [1]. This term was first used by Paniker and co-workers in the 1970s in a study on the levels of the reduced and oxidised form of glutathione (GSH/GSSG) in normal human erythrocytes treated with hydrogen peroxide as compared to erythrocytes deficient in glutathione reductase [2]. Initially, oxidative stress was defined in 1985 as a state, in which the overproduction of ROS, being the consequence of cellular respiration, the result of an acute inflammatory response or exogenous factors stimulating their production (e.g., environmental pollution), exceeding the ability of the organism’s endogenous antioxidant system to neutralise these oxidants [1]. In later years, as new discoveries demonstrated the inefficacy of antioxidant-based therapies on the one hand [3,4,5,6] and the physiological roles of ROS on the other [7], the research interests shifted from ROS-induced damage of biomolecules to redox signalling and regulation. The understanding of oxidative stress was evolving in parallel and currently, it is defined as “an imbalance between oxidants and antioxidants in favour of the oxidants, leading to the disruption of redox signalling and control and/or molecular damage” [8].

The antioxidant system of the human body consists of antioxidant enzymes and non-enzymatic low-molecular weight compounds with reducing properties. Among the enzymes, the main roles in the protection against ROS play superoxide dismutases (SOD1-3), catalase (CAT), glutathione peroxidase (GPx), peroxiredoxins (PRDXs) and sulfiredoxin (SRXN1). The latter enzyme is indispensable for the reactivation of some PRDXs if they become inactivated under conditions of oxidative stress [9]. The non-enzymatic and most abundant low-molecular-weight reducing compounds include uric acid, melatonin, bilirubin, lipoic acid, and the most important of them – glutathione that is present in cells in concentrations reaching even millimolar levels [10]. The knowledge of the key role of oxidative stress in the aetiology of civilisation diseases has resulted in the common belief that ROS are only harmful to human health. The reasoning was that uncontrolled exposure of cells to ROS causes protein oxidation leading to, for instance, the inactivation of key enzymes, contributing to lipid peroxidation impairing the functions of the cell and organelle membrane, which may also initiate promutagenic damage to nucleic acids [11]. Simultaneously, it was presumed that if oxidative stress resulting from the weakening antioxidant barrier of the body promotes the development of disease then exogenous agents neutralising ROS, such as plant-derived antioxidants, should be able to counteract, slow down development, or even support the treatment of diseases associated with oxidative stress.

## 2. Challenges Faced by Oxidative Stress Hypothesis

**Figure 1:**
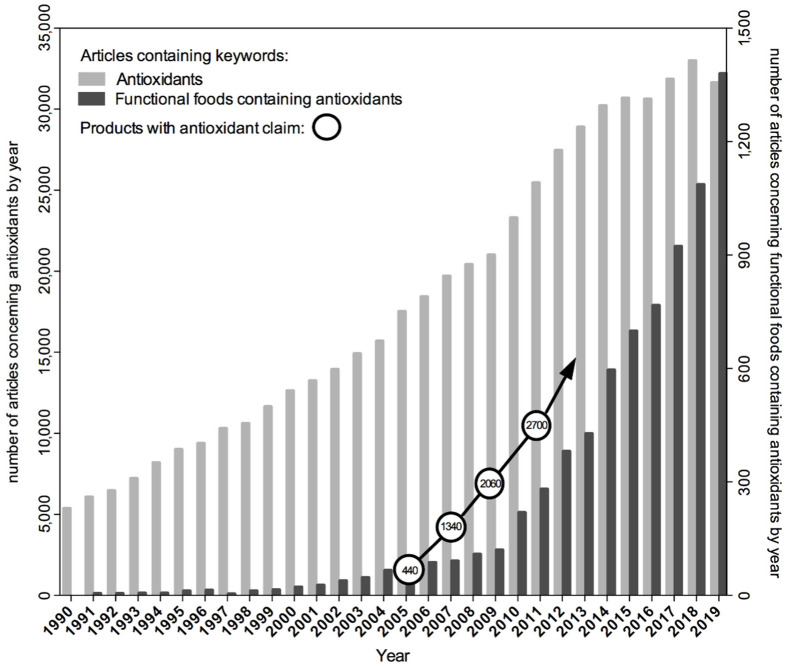
The number of scientific articles published in the PubMed database in 1990–2019 with the term “antioxidant” and “functional food containing antioxidants” among the keywords compared to the number of products bearing information about the presence of antioxidants that were introduced to the market in 2005–2011 (on the basis of [12]).

Formulation of the hypothesis suggesting the causative role of oxidative stress in disease initiated its verification by scientists around the world with the use of a variety of in vitro and in vivo research models and by designing studies with the participation of volunteers or conducting population observations. Intervention studies tested the effect of dietary supplements containing plant antioxidants isolated from natural sources, especially β-carotene and vitamins A, C and E, in the prevention of chronic diseases. However, this hypothesis also aroused the wide attention of the media, as well as the food and supplement industry, well before the research results became available and the assumptions confirmed. Manufacturers started to use the term “antioxidants” as an advertising slogan for their products. It has also become a common practice to formulate dietary recommendations promoting the increased consumption of antioxidants, the source of which can be not only natural plant-based foods but also dietary supplements. At that time, the latter were gaining popularity at a pace. The number and variety of antioxidant products marketed after 2005 began to grow much faster [12] than the number of scientific articles published in the PubMed database with the term “antioxidant” among the keywords (Figure 1). As a consequence, the commercial success of antioxidants preceded research on understanding their real effects on the body’s redox homeostasis.

The emerging results of research on the influence of antioxidants on the redox homeostasis of the organism turned out to be not as unambiguous and promising as initially assumed. One of the biggest disappointments in chemoprevention research was a project on the use of retinol precursors in the prevention of lung cancer. Epidemiological data and in vitro studies pointed to the association between low vitamin A levels in the body and the increased probability of developing the disease [13]. These observations led to the assumption that β-carotene supplementation in the case of cigarette smokers could prevent the development of lung cancer. To confirm this hypothesis, two series of clinical trials were undertaken. The first one, known under the acronym ATBC trial (Alpha-Tocopherol and Beta-Carotene), was conducted in Finland and involved administering β-carotene, vitamin E, their mixture or a placebo to 29,133 smokers at the age of 50–69. The alarming results obtained 5 years after the start of the supplementation caused the immediate termination of this study. It turned out that dietary supplementation with β-carotene increased the risk of lung cancer by 18% [14]. In the second project, known under the acronym CARET (The Beta-Carotene and Retinol Efficacy Trial) carried out in the USA, the effect of supplementation with β-carotene and retinyl palmitate on the incidence of lung cancer in high-risk populations, namely in cigarette smokers or people exposed to asbestos, was investigated. The observations indicated that as a result of this intervention, the risk of lung cancer in the smoking group increased up to 25% [4]. However, when other biological activities were considered, other studies revealed the beneficial effects of β-carotene consumption. It turned out that long-term intake of β-carotene was associated with slowed-down cognitive degeneration with age in the group of men participating in this trial [15]. Another example of unfulfilled expectations was the study on the chemopreventive activity of folic acid. Data published in 2007 showed that administration of folate before the onset of neoplastic lesions may prevent cancer development, but when folate delivery takes place after the onset of early lesions, supplementation appears to accelerate the tumorigenic process [16].

Another compound, whose preventive properties were studied extensively, was vitamin E. In 1992, Lee and co-workers began research to determine the effect of vitamin E supplementation on the reduction of risk of cardiovascular disease and cancer in healthy women. The results of these studies indicated that the intake of vitamin E every other day for about 10 years did not protect against cardiovascular disease or cancer but reduced mortality from cardiovascular disease in healthy women [17]. However, neither of these studies took into account the possible benefits of vitamin E in the subgroups with increased levels of oxidative stress in the body. Such a group may include people suffering from type 2 diabetes [18]. In the case of patients with type 2 diabetes, the particular type of haptoglobin (Hp), an antioxidant protein, is an indicator of cardiovascular problems. The gene encoding this protein is polymorphic and there are two alleles of this gene—1 and 2. The allelic protein product Hp 2 provides an inferior antioxidant barrier compared to the allelic product Hp 1. The research group led by Levy assumed that vitamin E can reduce cardiovascular problems in people with type 2 diabetes with the Hp 2-2 genotype, i.e., a subgroup comprising 2–3% of the general population. Indeed, it was confirmed that supplementation with vitamin E reduced the risk of myocardial infarction by 43% and deaths from cardiovascular diseases by 55% among people with type 2 diabetes with the Hp 2-2 genotype [18].

The extensively investigated and yet also rather disappointing example of outcomes of antioxidant-based interventions is the prevention and/or co-treatment of cancer with vitamin C. The pioneer in this area was McCormick, who in the 1950s proposed that insufficient amounts of vitamin C, resulting in weakened collagen synthesis and degeneration of connective tissue, could facilitate cancer metastasis [19]. In the 1970s, the concept of vitamin C's utility in cancer treatment was further extended in clinical trials by Cameron, who initially collaborated with Campbell and then with Pauling. With Pauling, he published results of two retrospective clinical trials, which reported that administration of high doses of vitamin C to patients affected by different, advanced cancers resulted in increased survival and alleviated symptoms [20,21]. These studies were criticised mainly for their retrospective nature and lack of blinding. Shortly thereafter, properly designed, randomised, controlled clinical trials with double-blinding conducted at the Mayo Clinic failed to provide evidence on the efficacy of high doses of vitamin C in the treatment of different types of cancer [22,23]. Despite methodological differences, there was one more factor, which at that time was not taken into account: distinct pharmacokinetics of orally and intravenously administered vitamin C, which in the case of the latter, can reach plasma concentrations exceeding those achieved by oral supplementation even up to 10-fold [24]. This led to the formulation of a new hypothesis that only *pharmacological* doses of vitamin C (i.e., those achieved following intravenous administration) could be considered as potential anticancer therapy. This idea was supported by mechanistic evidence indicating that high doses of vitamin C can induce oxidative stress in cancer cells through *H_2_O_2_* formation in the course of the Fenton reaction. Nevertheless, despite successful interventions in preclinical models, the evidence of its efficacy from clinical studies is still limited [19]. Currently, the interest in vitamin C-based cancer therapies has re-emerged mainly due to discoveries of its role as a cofactor in epigenetic enzymes: the ten-eleven translocation (TET) methylcytosine dioxygenases involved in the process of DNA demethylation and Jumonji-C domain-containing histone demethylases (JHDM) [25]. Epigenetic alterations, such as aberrant methylation of DNA and histones, can affect the expression of, e.g., tumour suppressor genes and oncogenes, which finally contribute to the process of tumo rigenesis. The reversible nature of epigenetic modifications has opened new and exciting ideas for cancer treatment, including the search for potential epigenetic modulators (such as vitamin C) [26].

Despite the mostly disappointing results of antioxidant vitamin-based interventions, the food, cosmetics and dietary supplement industries have not been discouraged from mass production and sale of products advertised under the pro-health slogan justified by the presence of antioxidants in their composition.

## 3. From Oxidative Stress to Reductive Stress

Progress in research on the cellular functions of ROS has gradually changed their only negative perception. The development of research technologies enabled more in-depth comprehension of the interactions between ROS and specific biomolecules. This contributed to the progress in the understanding of the role of individual oxidants not only in the aetiology of chronic diseases but also in physiology, including the regulation of metabolism, proper functioning of the nervous, cardiovascular and immune systems as well as in ageing. It turned out that participation in signalling pathways, which stimulate or silence the expression of specific genes, makes ROS a key factor in the regulation of cellular processes such as proliferation, differentiation, migration and cell death [27]. Comprehension of ROS impact on cellular functions has led to the conclusion that both the excess of antioxidants in the diet and the overactivity of the cellular antioxidant system are not beneficial for human health and the latter can lead to the so-called *reductive stress* [28].

Contrary to oxidative stress, reductive stress is not a well-established concept [29]. This term was first used by Gores in 1989 while reporting results of the study, in which hypoxia was mimicked by treating the rat hepatocytes with chemicals that block mitochondrial respiration and ATP production (chemical hypoxia) [30]. These authors observed that the electron carriers were reduced due to the limited availability of oxygen and then were re-oxidised during re-oxygenation, which led to a respiratory burst, i.e., the sudden production and release of ROS. Currently, reductive stress is defined as a pathophysiological situation, in which the cellular oxidoreductive balance is shifted towards an increased reduction state through the accumulation of endogenous antioxidants (e.g., GSH), reducing equivalents (such as NAD(P)H/NAD(P)^+^), or exogenous antioxidants, which affect cellular control or regulation of redox-related processes through the Nrf2–Keap1 system and other redox-related factors, as described in the next chapters [31,32,33].

### 3.1. Cellular Redox Buffers

**Figure 2:**
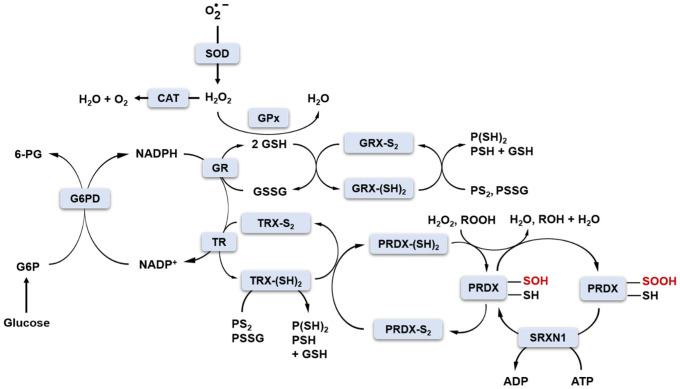
The balance between oxidants and antioxidants ensures cellular redox homeostasis. The abbreviations used relate to: 6-PG—6-phosphogluconate; ADP—adenosine 5′-diphosphate; ATP—adenosine 5′-triphosphate; G6P—glucose-6-phosphate; G6PD—glucose-6-phosphate dehydrogenase; GPx—glutathione peroxidase; GR—glutathione reductase; GRX—glutaredoxin; GSH—glutathione; GSSG—GSH disulfide; *H_2_O_2_* —hydrogen peroxide; NADPH—reduced NADP^+^; $O_{2}^{•\;-}$—superoxide radical; PRDX—peroxiredoxin; PSH—protein-glutathione; PSSG—protein-glutathione disulfide; SOD—superoxide dismutase; SRXN1—sulfiredoxin 1; TR—thioredoxin reductase; TRX—thioredoxin [illustration based on work of Xiao and Loscalzo [34] with modifications].

Under physiological conditions, cellular oxidoreductive homeostasis is maintained by redox buffers consisting mainly of GSH/GSSG, NAD(P)H/NAD(P)^+^, thioredoxins TRX1-2 and redox enzymes (SOD1-3, GPx1-8, glutathione reductase GR, glutaredoxins GRX1-2, peroxiredoxins PRDX1-6, sulfiredoxin SRXN1, thioredoxin reductases TRs, CAT), which have sufficient “buffering capacity” to keep the proportions of cellular oxidants and reductants at the correct physiological level (Figure 2) [34].

The production of cellular oxidants is compartment-specific, but molecular targets may be local and/or distant from their production site. For instance, *H_2_O_2_* generated by mitochondria can act as a mitochondrial and/or cytosolic signalling molecule. Unlike oxidants, antioxidant enzymes perform their functions in specific subcellular locations. For example, SOD2, an enzyme located in the mitochondrial matrix, can only convert $O_{2}^{•\;-}$ to *H_2_O_2_* in the mitochondria, where *H_2_O_2_* is then reduced to water by mitochondrial peroxidases such as CAT, GPx1, GPx4, and PRDX2 [35]. For that reason, to be in control of redox homeostasis, families of antioxidant enzymes such as SOD, GPx or PRDXs consist of different subclasses/members sharing a common mechanism of action but expressed in different cellular locations.

The GSH/GSSG and NADP^+^/NADPH redox couples play a key role in the two-electron reduction of peroxides (Figure 2). NADPH is produced by glucose-6-phosphate dehydrogenase (G6PD) in the pentose phosphate pathway (PPP) of glucose metabolism. GSH is a co-substrate for GPx in the reaction of *H_2_O_2_* reduction. In this reaction, two molecules of GSH donate two electrons to reduce *H_2_O_2_* to H_2_O and become oxidised to GSSG. The GSH pool is restored by glutathione reductase (GR) and NADPH as an electron donor (Figure 2) [36]. Glutaredoxins (GRX1-2) also use GSH as a cofactor to reduce protein disulfide bonds or protein-glutathione disulfides (PS2 or PSSG, Figure 2) [37]. NADPH is an essential cofactor not only in GR-catalysed reactions but also in the action of thioredoxin reductases (TRs). In this case, NADPH also provides two electrons for TR to reduce the oxidised TRX (TRX-S_2_) to the active form containing the sulfhydryl groups TRX-(SH)_2_. The reduced form of TRX-(SH)_2_ serves as an electron source for the regeneration of PRDXs I-V (PRDX-S_2_ to PRDX-SH). PRDXs are ubiquitous antioxidant enzymes that reduce *H_2_O_2_* and other organic hydroperoxides to water and alcohol, respectively [34,38]. Mammalian PRDXs are divided into three subgroups (typical 2-Cys, atypical 2-Cys and 1-Cys) based on the presence and location of the conserved resolving Cys (C_R_) residue. Typical 2-Cys PRDXs possess in their catalytic centre both C_R_ and peroxidatic Cys (C_p_). Upon reaction with peroxides, C_p_-SH can become oxidised initially to C_p_-SOH sulfenic acid form, which can form an intermolecular disulfide bond with C_R_-SH residue [9]. The C_p_-S-S-C_R_ bond can be further reduced by NADPH-dependent TRX, as already mentioned. The large distance between C_p_ and C_R_ (~13 Å), as well as the presence and interactions between specific structural motifs in the catalytic site of 2-Cys PRDXs, were proposed as grounds for the increased susceptibility of C_p_-SOH to further oxidation to C_p_-SO_2_H sulfinic acid or C_p_-SO_3_H sulfonic acid forms due to too slow formation of disulfide bond [39]. Such hyperoxidation results in the inactivation of PRDXs and loss of antioxidant activity. The physiological role of such inactivation is to allow for the accumulation of *H_2_O_2_* for signalling purposes (floodgate theory) and/or to gain chaperone function independent of peroxidase activity [39]. Hyperoxidation to the C_p_-SO_3_H sulfonic acid form is irreversible; however, C_p_-SO_2_H sulfinic acid can be reduced back to the active form with the aid of ATP-dependent sulfinic acid reductase, SRXN1. SRXN1 can restore the activity of only one subgroup of PRDXs, i.e., typical 2-Cys PRDXs [40]. The proposed mechanism of SRXN1-driven reactivation of PRDXs involves ATP-mediated phosphorylation of C_p_-SO_2_H and formation of a phosphoryl sulfinyl anhydride intermediate (PRDX-C_p_-SO_2_PO_3_^2-^) [41]. Subsequently, a thiosulfinate is formed upon the attack of conserved Cys residue in SRXN1 (PRDX-C_p_-SO-S-Cys-SRXN1). The collapse of this intermediate, release of PRDX in C_p_-SOH form and its further reduction to C_p_-SH is mediated by thiols such as GSH or TRX [42]. It is postulated that protection of cells against oxidative damage via maintaining the activity of PRDXs is the major role of SRXN1. In the research conducted by our group (article under review), we also proposed that downregulation of the expression of this antioxidant enzyme may serve as a fine-tune regulation of redox homeostasis preventing the occurrence of reductive stress induced by the presence of high concentrations of strong reducing agents, such as catechins. Nonetheless, exogenous antioxidants provided with a daily diet may be needed to support endogenous antioxidant systems when challenged by exogenous oxidative stress inducers.

### 3.2. Health Risks Related to Disrupted Redox Balance

Even though antioxidant consumption has been repeatedly associated with a decreased risk of certain diseases (such as cardiovascular, neurodegenerative diseases or cancer) [43,44], it should not be forgotten that excessive intake of antioxidants may carry certain risks as well [45]. Both excessive levels of oxidants and reducing agents, i.e., exceeding the buffering capacity of the cells, can lead to the disruption of the redox balance and result in oxidative or reductive stress, respectively, with the latter being disproportionately underinvestigated. The reductive stress may cause the neutralisation of ROS in cells to a level below physiological concentrations, which disturbs their signalling functions. Therefore, an excess of reducing factors weakens cell growth, interferes with mitochondrial function and cellular metabolism, and induces changes in the disulfide bond profile in proteins [46]. For example, the endoplasmic reticulum (ER) functions in a relatively oxidising environment that is necessary to maintain the disulfide bonds in membrane and secretory proteins. Under reductive stress, the reduction of disulfide bonds to sulfhydryl groups takes place. This in turn results in the activation of unfolded protein response and ER stress [34]. The results of studies, involving mice with cardiac-specific overexpression of mutated human aB-crystallin (hR120gCryAB), revealed elevated GSH levels and increased activities of GR, G6PD, CAT and GPx, thus demonstrating that reductive stress can be dangerous to health. The detected changes indicated the emergence of hypertrophy and heart failure in these mice [47,48]. For reviews on the role of reductive stress in cardiovascular pathologies, the reader should refer to the following articles: [49,50].

#### 3.2.1. Diabetes

In animals with early diabetes, metabolic disruptions were shown to elevate the ratio of cytosolic NADH/NAD^+^ and therefore to disturb redox homeostasis towards reductive stress [51]. Excessive amounts of glucose (hyperglycemia) initially contribute to reductive stress due to the overproduction of NADH and thus, disrupt the NADH/NAD^+^ ratio [52]. In the course of glucose catabolism, NADH is first generated by glyceraldehyde 3-phosphate dehydrogenase during glycolysis and then, upon conversion of pyruvate into acetyl-CoA by pyruvate dehydrogenase. When acetyl-CoA enters the Krebs cycle, NADH is generated by the other three enzymes: isocitrate dehydrogenase, α-ketoglutarate dehydrogenase and malate dehydrogenase. Another source of excessive NADH generation is the polyol pathway, which is active mainly during hyperglycemia due to the high K_M_ value for aldose reductase (70 mM) [53]. In the course of this pathway, aldose reductase converts glucose into sorbitol, which is subsequently oxidised to fructose by sorbitol dehydrogenase with the concomitant reduction of NAD^+^ to NADH. This contributes to a further increase in the NADH/NAD^+^ ratio and thus, under hyperglycemic conditions, complex I in the electron transport chain (ETC) is overburdened with the recycling of NADH back into NAD^+^. Upon increased proton pumping through complex I, the electron leakage is enhanced, which facilitates subsequent partial reduction of oxygen and leads to overgeneration of $O_{2}^{•\;-}$ and other forms of ROS [54]. Thus, reductive stress turns out to be an intermediate state preceding the occurrence of oxidative stress.

#### 3.2.2. Alzheimer’s Disease

With the development of knowledge on the aetiology and course of chronic non-infectious diseases, it has become evident that both states of disturbed redox homeostasis, including oxidative and reductive stress, are interdependent. The boundary between the occurrence of these pathological states can be very difficult to define due to their dynamics and the broad spectrum of redox reactions taking place in cells [55]. Paradoxically, reductive stress may promote the production of ROS (e.g., by partial reduction of molecular oxygen to radical forms), and as a result, may initiate the processes leading to oxidative stress. Alzheimer’s disease (AD) vividly illustrates how the modulation of redox status may impact the development of chronic diseases. In the case of healthy people with a genetic risk predisposing to the development of this disease, which is the e4 variant of the *APOE* gene, the state of reductive stress was observed many years before the development of this disease [56,57]. One of its markers was the increased level of glutamylcysteine ligase, an enzyme involved in the antioxidant cellular defence against oxidants in healthy people at risk of AD compared to the control group. However, in patients with developed AD, the level of this enzyme was lower than in the control group. Additionally, in healthy people at risk of developing AD, a decrease in the level of two markers of oxidative stress (MAP-p38 kinase phosphorylation and GSSG concentration) was observed, while in patients suffering from AD, the level of these factors was significantly increased [56]. This suggests that along with the progression of the disease, the redox status changed from reductive to oxidative. However, the exact mechanism of this phenomenon has not been revealed yet. In the case of people with an increased risk of developing AD, the continuous production of small amounts of oxidants was observed. This persistent production of oxidants could result in the hormetic overexpression of antioxidant enzymes. In turn, the stimulation of the endogenous antioxidant system by ROS leads to the shift of the redox balance of the cells towards reductive stress. The overactivity of antioxidant mechanisms may become suppressed later in life. In addition, along with the development of the disease, among other pathologies, β-amyloid deposition in the brain tissue is observed. These deposits are formed when the amyloid precursor protein APP is cleaved by enzymes from the secretase family. Under normal conditions, fragments of β-amyloid undergo further degradation but not in AD patients, especially not in those with the *APOE4* genetic variant. Apolipoprotein increases the breakdown of β-amyloid; however, some of its isoforms, such as APOE4, do not effectively complete this task. Unremoved β-amyloid increases mitochondrial peroxide production, protein nitration and oxidation, and cytochrome *c* release [58]. The continuous production of radicals resulting mainly from the pathological deposition of β-amyloid in the brain tissue ultimately leads to oxidative stress in neurons and the appearance of clinical symptoms of the disease [56].

AD serves as an example of a condition where both redox imbalances can have a critical impact on human health. Supplementation with exogenous antioxidants in the case of healthy people, but at increased risk of developing AD, could be associated with the risk of exacerbated reductive stress, which in turn might affect the further course of the disease. In such a situation, the prophylactic use of antioxidants without the knowledge about redox status could be simply harmful. Unfortunately, diagnosis of people at risk of AD is difficult and becomes often possible only when the first clinical symptoms of the disease appear. Then, however, when the redox status shifts towards oxidative stress, the additional consumption of antioxidants could have positive effects. Recent research has shown that increased consumption of coffee, a product rich in bioactive phytochemicals such as caffeine and polyphenolic antioxidants, including chlorogenic acid or flavonoids, contributes to the reduction of the pathological deposition of β-amyloid in the brain, which in turn slows down the deterioration of cognitive functions [59].

#### 3.2.3. Ageing

Another example illustrating the balancing of the organism between reductive and oxidative stress is the study on the influence of hyperbaric oxygen therapy on ageing. At the cellular level, the shortening of the telomere length is one of the key hallmarks of ageing processes. While many genetic and environmental factors can influence telomere attrition, oxidative stress appears to be one of the most important. Telomeric sequences are rich in guanines and therefore very sensitive to oxidative damage that causes the shortening of telomeric DNA if the DNA repair by the base excision repair is unsuccessful [60]. *In vitro* studies consistently indicate that in cellular models even mild oxidative stress accelerates telomere shortening, while antioxidants reduce the rate of this process and extend cell viability [61]. It is known that exposure of cell cultures to a hyperbaric environment causes oxidative stress and premature cell ageing. Surprisingly, the elongation of telomeres in leukocytes was observed in divers exposed to hyperbaric oxygen [62]. Repeated intermittent exposures to hyperoxia, e.g., using hyperbaric oxygen therapy, increased the telomere length of peripheral blood mononuclear cells by more than 20%. Further studies showed that intermittent hyperoxic exposures by inducing increased ROS production resulted in the increased production of endogenous antioxidants and stimulation of the expression of genes encoding proteins that make up the body’s antioxidant system [63]. Single exposures rapidly increase the production of ROS, triggering a sudden antioxidant response. After repeated exposures, this reaction becomes permanent and thus provides better antioxidant protection, at least over telomeric DNA.

Similarly to oxidative stress, reductive stress is also associated with some pathological conditions [34,64]. The current knowledge clearly revealed the pleiotropic activity of ROS and brought about the understanding that the formerly suggested risks do not result from the mere presence of ROS, but rather from the disturbance of their level, either being increased or decreased. These impairments trigger a network of redox-dependent signalling pathways to restore normal redox homeostasis [55,65]. Nowadays, research on redox signalling and redox-active components forming redox networks has become a very active area of biological investigations.

## 4. Redox Network

The oxidoreductive reactions that are the basis of mitochondrial respiration are essential for energy generation and are the most important mechanisms ensuring the proper function of cells, tissues, and ultimately the entire body. The by-products of the energy-generating processes are ROS, i.e., molecules responsible for the control of cellular redox signalling described in the previous section. Thus, the amount of ROS may be considered as a kind of indicator of the energy state of the cell. A healthy person at rest produces as much ATP per day as approximately her or his body weight. With increased physical activity, this amount can rise up to 0.5 kg per minute. Since the body cannot store ATP, the mitochondria must function continuously. The average cell uses 10 billion ATP molecules daily, which means that an adult produces 3.0 × 10^25^ ATP molecules every day, thus equivalent to approximately 1200 watts of energy [66]. The flow of electrons through the ETC leads to the reduction of molecular oxygen to water in the reaction catalysed by cytochrome *c* oxidase (complex IV). However, some of the electrons leak out from the ETC instead of being used for this reaction. As was already described in the case of hyperglycemia, the more intensive the synthesis of ATP is, the greater the number of electrons that may escape. These electrons are then utilised in the reaction not controlled by complex IV, namely for the reduction of oxygen to $O_{2}^{•\;-}$. The generated radicals undergo disproportionation to *H_2_O_2_* and O_2_ in a reaction catalysed by SODs. Oxygen is then recycled to the ETC, while *H_2_O_2_* is the main molecule used in redox signalling.

### 4.1. Sources of ROS

At rest, the body uses 1 kg of oxygen daily. Approximately 1–2% of oxygen is “lost” in the mitochondria as a result of electron leakage from the ETC [67]. Assuming a constant energy turnover by the mitochondria, the ETC may be regarded as the main source of *H_2_O_2_* and other cellular ROS. In addition to the natural leakage of electrons from the ETC, which is specific for a given cell type and correlates with the metabolic rate, there is also an induced dissipation of protons from the mitochondrial intermembrane space, by the action of, e.g., uncoupling proteins (UCP). These proteins are involved in a process called thermogenesis. Under normal conditions, the activity of UCP1 is inhibited by purine nucleotides, e.g., ATP. As the body temperature lowers, the nerve impulse is transferred from the brain through the sympathetic nervous system to brown adipose tissue. Then, norepinephrine is released. This neurotransmitter binds to the β_3_-adrenergic receptors of adipocytes, which stimulates adenylate cyclase to produce cyclic adenosine monophosphate (cAMP). The appearance of cAMP stimulates protein kinase A (PKA) that through phosphorylation activates lipases hydrolysing triacylglycerols to free fatty acids. The released fatty acids counteract the inhibition of UCP1 by ATP. UCP1 protein is located in the inner membrane of mitochondria present in brown adipose tissue and has the form of an ion channel [68]. It dissipates the electrochemical proton gradient, thus causing decline in the proton-motive force and ATP production. The passage of protons from the mitochondrial intermembrane space to the mitochondrial matrix through this channel does not generate ATP, as in the process of oxidative phosphorylation, but produces heat energy, which is a key principle of thermogenesis in brown adipose tissue. The mentioned decrease in proton-motive force across the inner mitochondrial membrane also reduces the production of $O_{2}^{•\;-}$ in the ETC, as demonstrated by Oelkrug et al. in 2010 [69]. *In vitro* studies have also shown that the use of UCP inhibitors, such as purine nucleotides, increased the electrochemical gradient and the concentration of $O_{2}^{•\;-}$ in the mitochondrial matrix [70]. Thermogenesis in brown adipose tissue is thus another mechanism of the ETC endogenous regulation of redox homeostasis.

It was shown that in myoblasts, about 45% of the cellular *H_2_O_2_* production comes from the ETC but it is not the only source of this ROS. About 40% is produced in reactions catalysed by NADPH oxidases (NOX) and the rest in other enzymatic processes. It follows that in the case of myoblasts, the contribution of NOX and the ETC to the production of *H_2_O_2_* is comparable. Nevertheless, the contribution of various mechanisms feeding the ROS pool depends on the cell type and its metabolic state [71]. In the case of transmembrane proteins from the NOX family, the cellular localisation and the level of ROS production vary significantly. In addition, NOX can also be bound to the membrane of specific, redox-active endosomes called redoxisomes [72], which are formed in response to extracellular stimuli such as nutrients, growth factors, or cytokines. Redoxisomes enable *H_2_O_2_* compartmentalisation for local regulation of redox balance or cell signalling. In addition to NOX and the ETC, *H_2_O_2_* is also generated by other oxidases present in subcellular locations, including the endoplasmic reticulum (ER) and peroxisomes as well as by the previously mentioned family of superoxide dismutases (SOD1-3). Another source of ROS is the catabolism of purines to uric acid. The final reactions are catalysed by xanthine oxidase, which uses oxygen as an electron acceptor, reducing it to $O_{2}^{•\;-}$, a radical that is dismutated to *H_2_O_2_* [73].

A total of 41 enzymes generating *H_2_O_2_* and/or $O_{2}^{•\;-}$ as side products were identified in human cells [74]. This list becomes much longer when enzymes that produce other ROS precursors, such as lipid hydroperoxides, nitric oxide (NO), and hypochlorous acid are considered. In addition to intracellular processes, oxidants are also generated in response to environmental factors, which are referred to as the exposome. This term embraces a set of molecular factors such as nutrients and non-nutrients from foods, drugs and toxins as well as physical agents (e.g., UV radiation, X-rays). There is also a growing body of evidence that the induction of ROS may be a consequence of psychological stressors such as permanent stress [75]. Therefore, there are many processes that are a potential source of ROS that affect the body’s redox signalling.

### 4.2. Standard Reduction Potentials (E^0^) of Proteins Shape Cellular Thiolstat

**Figure 3:**
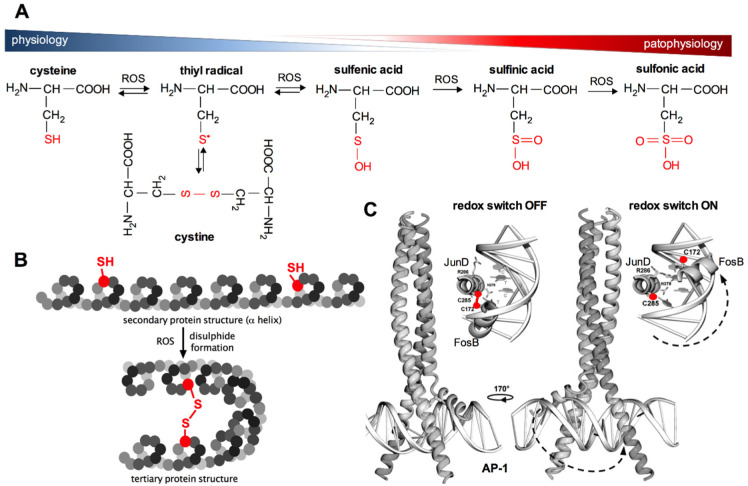
Oxidation of the sulfhydryl group of cysteine. Panel (**A**)—physiological (disulfide, sulfenic acid) and pathophysiological (sulfinic and sulfonic acid) products of cysteine oxidation. Panel (**B**)—the effect of disulfide bond formation on protein α-helix conformation. Panel (**C**)—mechanism of redox regulation on the example of the transcription factor AP-1 (activating protein 1). The enlarged view shows the DNA binding units (JunD and FosB) of AP-1. The disulfide bond between JunD and FosB (redox switch OFF) must be reduced to sulfhydryl groups to allow FosB subunit to bind to DNA (redox switch ON). Disulfide reduction is accompanied by conformational changes (dashed lines) [computer visualisation adopted from Yin et al. [78].

In organisms, the formation of ROS during oxidative respiration is primarily utilised for controlling biological processes such as cell growth and division, which are absolutely dependent on the availability of energy resources. However, ROS are also major signalling agents affecting specific molecular targets, thereby helping cells to adapt to a changing environment. Redox signalling is mainly related to the regulation of the so-called thiol redox status by influencing the structure of proteins, which determine their function. This specific physiological mechanism of the regulation of cellular functions through redox reactions of thiol groups in proteins was named “cellular thiolstat” by Jacob in 2011 [76]. This mechanism decides about the type of the transmitted signal, enzyme activity or gene transcription. It was shown that about 10–20% of proteins in the cellular proteome are easily oxidised under aerobic conditions. The thiolstat system is based on gradual cysteine oxidation, which enables the cell to appropriately react to changes in the intracellular redox environment in a thermodynamically controlled and reversible manner. Firstly, the gradual changes of the cellular thiolstat are possible because cysteine residues in proteins have different values of standard reduction potentials (*E^0^*), therefore they show different susceptibility to oxidation depending on the composition of amino acids in the surrounding polypeptide chain [76]. Secondly, oxidation of the cysteine sulfhydryl group can lead to the formation of a number of oxidation products (Figure 3A). Cellular redox homeostasis is highly dependent on the form of cysteine residues found in proteins. Disulfides are the most common products of oxidation of sulfhydryl groups under physiological conditions and under mild oxidative stress (S-thiolation, S-glutathiolation, formation of intra- and intermolecular disulfide bonds, Figure 3). The formation of disulfide bonds may be accompanied by conformational changes in the protein structure, which determines its final conformation and thus its biochemical activity (Figure 3B,C). Under conditions of increased oxidative stress, the sulfhydryl groups of cysteines are oxidised in the first step to sulfenic acids and finally to sulfinic and sulfonic acids (as in the case of peroxiredoxins). The greatest threat to cells resulting from oxidative stress is the risk of irreversible oxidation of the -SH groups of proteins to sulfinic and sulfonic acids (Figure 3A), which may cause the impairment of biological functions dependent on the nucleophilic properties of –SH groups in proteins [76]. Importantly, there is an exception to this rule, as described earlier, since overoxidation of -SH group to sulfinic acid in 2-Cys PRDXs can be reversed with the aid of SRXN1 [77].

### 4.3. Redox Regulation of Protein Kinase Activity

A common cellular thiolstat-dependent regulatory mechanism is the modulation of protein kinase activity by redox modification of the cysteines [79]. These enzymes catalyse the phosphorylation of a protein specific to a given kinase. The reversible phosphorylation mainly of serine, threonine or tyrosine residues in proteins is one of the most important and well-studied post-translational modifications. It is estimated that one-third of the proteins in the human proteome are substrates for the phosphorylation reaction. The nucleophilic groups of the amino acids attack the end phosphate group of ATP (γ-PO_3_^2-^) forcing it onto the hydroxyl group in the side chain of amino acid [80]. This modification gives rise to conformational changes in the protein, modulating its function in two ways. In the first case, conformational changes determine the catalytic activity of the protein. Thus, the enzyme can be activated or inactivated by phosphorylation. In the second mechanism, the phosphorylated proteins recruit other adjacent proteins that have structurally conserved domains recognising phosphorylated residues and bind to them. The ability of phosphorylated proteins to recruit other proteins is critical for cell signal transduction. Thus, redox modulation of protein kinase activity plays a key role in the regulation of many cellular processes including the cell cycle, growth, apoptosis, and numerous signal transduction pathways.

The phosphorylation of protein tyrosine residues is influenced by the direct regulation of protein-tyrosine phosphatase and protein-tyrosine kinases, based on oxidation–reduction reactions. On the one hand, the oxidation of reactive cysteine in protein-tyrosine phosphatases causes their inactivation, resulting in an increased level of tyrosine phosphorylation. On the other hand, however, as discussed above, protein-tyrosine kinases can be activated by ROS (Figure 3). This is the case of EGFR (epidermal growth factor receptor), where oxidation of cysteine to sulfenic acid stimulates the activity of protein-tyrosine kinase of this receptor [81]. It was shown that growth factor receptors, frequently showing tyrosine kinase activity in their cytoplasmic domains, are overexpressed in many tumours, hence in the cells living under constant oxidative stress. Receptor overactivity makes the cell more sensitive to such amounts of growth factors that do not normally trigger proliferation. The sustained proliferative signalling is one of the main hallmarks of cancer that were proposed by Hanahan and Weinberg in 2000 and extended to further characteristic features of cancer in 2011 [82,83].

### 4.4. Thiol Redox Signalling in Metabolic Pathways—AMPK, GAPDH

Regulation of energy stress via AMP-activated serine/threonine kinases (AMPK) is also under redox control. Although AMPK contains cysteine residues in its structure that could provoke its susceptibility to thiolstat regulation, it has recently been shown that the activity of AMPK is not modulated by direct oxidation of these cysteines. Activation of AMPK appears to be a consequence of the influence of the cellular oxidoreductive status on mitochondrial respiration, whose marker is ATP. The decrease in ATP levels (and concomitant increase in AMP levels) results in the activation of AMPK [84]. AMPK phosphorylates a diverse set of proteins that redirect cellular metabolism towards ATP-generating pathways, such as fatty acid oxidation, autophagy, and glucose consumption, in order to restore the correct energy balance while restricting anabolic pathways that consume ATP (fatty acid, lipid and protein synthesis, gluconeogenesis, cell growth and proliferation) [85].

Another interesting mode of thiol redox signalling related to metabolism is the modulation of glyceraldehyde-3-phosphate dehydrogenase (GAPDH) activity, which is the major enzyme in the energy-producing stage of glycolysis. In the cytoplasm, glyceraldehyde-3-phosphate reacts with the ionised sulfhydryl group of Cys152 at the active site of GAPDH and becomes oxidised to carboxylic acid, while reducing NAD^+^ to NADH. The product of this reaction is a high-energy thioester. In the next step, NADH dissociates from the complex under the influence of another NAD^+^ molecule, while the thioester in reaction with orthophosphate forms an intermediate, which then decomposes to 1,3-bisphosphoglycerate and the enzyme. Thus, the sulfhydryl group Cys152 is crucial for the glycolytic activity of GAPDH [86]. The cellular energy abundance, as previously emphasised, is “communicated” by the by-products of cellular respiration, i.e., ROS. When the energy level is sufficient for the cell, ROS oxidise the Cys152 sulfhydryl group in the catalytic centre of this enzyme, blocking interaction with the glycolytic substrate, i.e., glyceraldehyde 3-phosphate. Oxidative modifications change the conformation of the enzyme, and hence its functions. It was shown that, as a result of oxidation, GAPDH loses its glycolytic functions, but may migrate to the nucleus where it is involved in various processes, including DNA repair [87].

### 4.5. Redox Modulation of Transcription Factor Activity

#### 4.5.1. Keap1-Nrf2 Pathway

**Figure 4:**
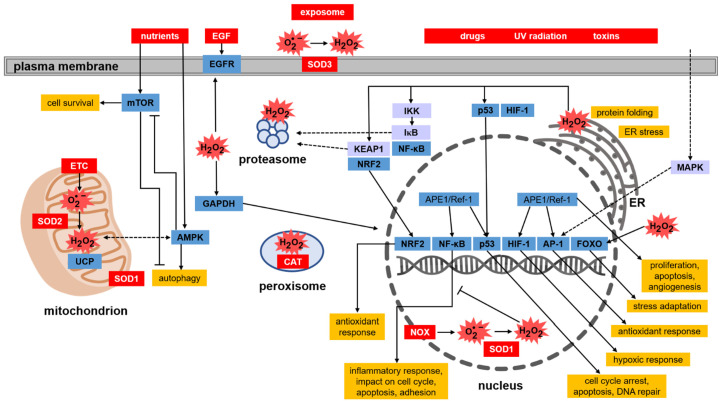
Signalling dependent on the redox status of the cell. Cellular enzymes constitutively or in response to exogenous triggers stimulate the production of ROS (red), which then affect various molecular targets (blue) modulating biological activities (yellow). The action of ROS is pleiotropic and includes the regulation of stress adaptation (including the antioxidant response), inflammatory response, cell death and metabolic adaptation (continuous arrows). A detailed description of the mechanisms is included in the text of chapter 2. The dashed arrows indicate intermediate processes. The abbreviations used refer to: AMPK—AMP-activated protein kinase; AP-1—activating protein 1; APE1/Ref-1—apurine/apyrimidine endonuclease1/redox factor 1; CAT—catalase; EGFR—epidermal growth factor receptor; ER—endoplasmic reticulum; ETC—Mitochondrial Electron Transport Chain; FOXO—transcription factor (forkhead box O); GAPDH—glyceraldehyde-3-phosphate dehydrogenase; GFR—epidermal growth factor; HIF-1—hypoxia-induced transcription factor; IκB—NF-κB inhibitor; IKK—NF-κB kinases; KEAP1—Nrf2 sensor/binding protein; MAPK—mitogen-activated kinases; mTOR—serine-threonine kinase, the so-called rapamycin mammalian target; NF-κB—nuclear factor-κB; NOX—NADPH oxidase; NRF2—nuclear factor 2; SOD1—3—superoxide dismutases; UCP—uncoupling protein.

The list of targets of thiol redox signalling is very wide and also includes the regulation of the activity of numerous transcription factors. The Keap1–Nrf2 pathway, however, deserves special attention (Figure 4). Protection against oxidative stress depends on the efficient endogenous system reacting to changes in ROS levels and the activity of antioxidant enzymes, whose expression is among others under the control of the Nrf2 factor (nuclear factor erythroid 2-related factor 2) [88]. When the oxidation–reduction potential of the cell is shifted towards oxidation, Nrf2 becomes activated, increasing the transcription of genes encoding phase II xenobiotic detoxification enzymes and antioxidant enzymes that contain the ARE (antioxidant response element) sequence in the promoter regions [89]. The transcription of genes under the control of Nrf2-ARE depends on many factors that impact the activity of Nrf2. One such factor is the Keap1 protein (Kelch ECH associating protein 1), which is an inhibitor of Nrf2 [90]. Keap1 contains 27 cysteine residues, which can be oxidised. However, the sulfhydryl groups of cysteines found only in the conserved polypeptide fragments such as Cys23, Cys151, Cys273, and Cys288 play a role in the interaction with Nrf2 [91]. Under proper redox homeostasis, the sulfhydryl groups of these cysteines remain reduced, which is necessary for the sequestration of Nrf2 from the cytoplasm and for its ubiquitination and further degradation in the proteasome. Under oxidative stress, the sulfhydryl groups Cys151 in two molecules of Keap1 become oxidised and connected by a disulfide bond, resulting in the formation of a Keap1 homodimer [92]. This leads to the loosening of Nrf2–Keap1 binding and finally to the release of Nrf2. Liberated Nrf2 translocates to the nucleus, where it serves as a transcription factor that triggers, after binding to ARE, the expression of a number of genes encoding proteins with antioxidant activity, mainly those involved in GSH-based redox systems (GPx, GR), TRX, PRDX, and SOD. Another mechanism influencing the activity of Keap1 is the phosphorylation and dephosphorylation of tyrosine at position 141. Keap1 after being synthesised de novo becomes phosphorylated at Tyr141. In this form, Keap1 is involved in the binding, ubiquitination, and degradation of Nrf2 [93,94]. Chemical or oxidative stress induces the dephosphorylation of Tyr141, which greatly reduces the stability and accelerates the degradation of Keap1, thus enabling the translocation of Nrf2 to the nucleus [92].

#### 4.5.2. NF-κB

Another important protein sensitive to thiol redox signalling is the nuclear transcription factor, namely NF-κB (nuclear factor-κB) [95]. This factor is involved in the body’s inflammatory response, cell cycle modulation, apoptosis, and cell adhesion (Figure 4). NF-κB dimers exist in the cytoplasm of most cells in an inactive form, because they are associated with protein IκB that blocks NF-κB activity. The complex with IκB prevents the translocation of the transcription factor from the cytoplasm to the nucleus. Under the influence of oxidants, mainly *H_2_O_2_*, appropriate kinases (IKK) are activated, which catalyse the phosphorylation of IκB [96,97]. In this form, IκB undergoes ubiquitination and then it is degraded in the proteasome. Released NF-κB dimers migrate to the nucleus where they induce the expression of genes under their control. Regulation of NF-κB by *H_2_O_2_* can also take place in the cell nucleus. Due to the presence of cysteines in the DNA-binding region of NF-κB, the increased level of *H_2_O_2_* in the nucleus causes oxidation of a cysteine residue (Cys62) in the NF-κB subunit p50, which disrupts the ability of NF-κB to bind to DNA. The transcriptional activity of sequences dependent on this transcription factor is thus blocked [97]. Under physiological conditions, thioredoxin (TRX) maintains Cys62 in the p50 subunit in a reduced state and hence, promotes NF-κB binding to DNA [98].

#### 4.5.3. p53

Another transcription factor regulated by the redox status of the cell is the p53 protein. It activates the transcription of genes involved in the cell cycle, DNA repair and apoptosis. All these processes are crucial for cell survival, therefore this protein is of particular interest in studies on the development and treatment of cancer. The transcription factor p53 has long fascinated and surprised researchers because of the wide range of cellular mechanisms, the course of which it may determine. Another reason for the popularity of p53 among researchers is the frequent mutations of the *TP53* gene observed in human cancers and the presence of many isoforms of this protein, often with still poorly understood functions [99]. Under physiological conditions, p53 is maintained at a low level in cells. This is due to the MDM2 protein (murine double minute 2), which by binding to p53, blocks its transcriptional activity and leads to ubiquitination-dependent degradation [100]. The formation of p53–MDM2 complexes lowers the level of free p53 in the cell nucleus. *MDM2* expression is induced by p53, which creates a self-regulating mechanism for the feedback control of cell proliferation. As a result of oxidative stress, p53 phosphorylation is catalysed by serine-threonine kinases, e.g., mitogen-activated kinase (MAPK). This modification blocks the interaction of MDM2 with p53, leading to the inhibition of MDM2-dependent degradation and, consequently, the accumulation of p53 in the cell nucleus. Additionally, there are 10 cysteine residues in the p53 amino acid sequence located in the DNA binding domain. This has an impact on the function of this transcription factor. *In vitro* studies have shown that the binding of p53 to DNA requires the availability of sulfhydryl groups in p53 and thus the reducing environment [101]. The redox effector factor 1 (Ref-1) was shown to stimulate p53 binding to DNA by maintaining the cysteines in the DNA-binding region in the reduced state [102] (Figure 4). Moreover, as in the case of NF-κB, p53 activity is dependent on the TRX and TR systems.

#### 4.5.4. AP-1

Both APE1/Ref-1, as well as the TRX and TR systems, are also involved in the activation of other transcription factors, e.g., AP-1 (activator protein-1), that are crucial for the regulation of cellular redox response [103]. The AP-1 protein regulates the transcription of genes associated with cell proliferation, differentiation and apoptosis [104]. This transcription factor is composed of nuclear proteins belonging to the Jun (c-Jun, JunB and JunD) and Fos (c-Fos, FosB, Fra-1 and Fra2) families. AP-1 forms heterodimers (Fos-Jun) or homodimers (Jun-Jun). In the DNA binding domains of this transcription factor, cysteines are present (Figure 3C and Figure 4) [104]. Oxidation of the sulfhydryl groups of these cysteines and the formation of disulfide bonds between AP-1 dimers prevents their binding to DNA, which blocks the transcription of genes controlled by this factor. The disulfide bond between the protein units in AP-1 dimers (JunD and FosB in Figure 3C) must be reduced to sulfhydryl groups in order to bind to DNA (Figure 3C). The reduction of the disulfide is accompanied by conformational changes. Under physiological conditions, the before-mentioned proteins, namely TRX and Ref-1, promote the binding of AP-1 to DNA by maintaining the sulfhydryl groups in the DNA binding domains in a reduced state [78].

#### 4.5.5. HIF-1α

The transcription factor, whose activity, in contrast, depends on the level of cellular oxidants, in this case, molecular oxygen, is HIF-1α (hypoxia-inducible factor-1α). This protein is the major regulator of the transcriptional response to decreased oxygen levels. Activation of the transcription factor HIF-1α is the best-known adaptive pathway in solid tumour cells growing in a hypoxic microenvironment. Under aerobic conditions, HIF-1α is not activated because two amino acids that are essential in its regulation (proline 402 and 564) are hydroxylated. HIF-1α modifying hydroxylases are oxygenases that require molecular oxygen as a substrate that is bound by Fe (II) found in their active site. Therefore, under conditions of reduced oxygen, this factor becomes activated. HIF-1α activates the transcription of more than 100 genes that regulate important biological processes necessary for the survival and progression of cancer cells [105]. Many recent studies have provided convincing evidence of a strong correlation between elevated HIF-1α levels and tumour metastasis, angiogenesis, treatment resistance, and consequently, poor patient prognosis.

#### 4.5.6. FOXO

Other redox-dependent transcription factors are proteins from the FOXO family (forkhead box, class O). These factors are important regulators of the cellular response to stress and enhance the cellular antioxidant defence. FOXO stimulates the transcription of genes encoding antioxidant proteins located in various subcellular compartments, for example in the mitochondria (SOD2, PRDX3, PRDX5) and peroxisomes (CAT) as well as antioxidant proteins found outside cells, e.g., in plasma (P-selenoprotein—SEPP1) [106]. Translocation between the cytoplasm and the cell nucleus regulates the activity of FOXO. When blood glucose is high, the pancreas releases insulin into the bloodstream. Insulin then activates phosphoinositide 3-kinase, which phosphorylates and thus activates the Akt protein kinase. Akt kinase in turn phosphorylates FOXO, which leads to cytoplasmic retention of this factor resulting in the inhibition of FOXO-dependent gene transcription [107]. In its unphosphorylated form, FOXO is localised in the nucleus where it binds to the insulin response sequence found in the promoter region of the glucose-6-phosphatase gene and activates its transcription. The increased levels of glucose-6-phosphatase accelerate the rate of glucose production in the liver [108]. This reaction completes the final stage of gluconeogenesis and therefore plays a key role in homeostatic blood glucose regulation. Alternatively, FOXO proteins may be phosphorylated by redox signalling dependent kinases, which leads to the accumulation of FOXO in the nucleus and the induction of the expression of genes encoding proteins involved in the stress response and enhancement of antioxidant protection. In this case, the oxidative stress-induced phosphorylation of FOXO is catalysed by the redox status-dependent JNK (c-Jun N-terminal kinases) [109].

## 5. Conclusions

The list of redox signalling targets is very long, and the examples presented in this review, although belonging to those most important, are only a fraction of the whole. The range of redox networks created by interactions of ROS with cellular molecules and the resulting regulation of processes at the genomic and proteomic levels confirms how important they are for the proper functioning of the organism. This specific “redox interactome” controls the coordination and synchronisation of processes that are the basis of life, such as energy-generating mechanisms. Since the regulation of processes engaged in the generation of energy is at the intersection of all metabolic pathways, redox processes are linked to most, if not all, physiological functions. Thus, oxidoreductive imbalances of the body, both those in favour of oxidation (oxidative stress) and excessive activity of the antioxidant system (reductive stress), may have a critical impact on human health. Scientists are strongly interested in the search for modulators of the redox status of cells due to the role of redox biology in the prevention of chronic diseases, especially cancer.
